# Mediodorsal Thalamus Is Critical for Updating during Extradimensional Shifts But Not Reversals in the Attentional Set-Shifting Task

**DOI:** 10.1523/ENEURO.0162-21.2022

**Published:** 2022-03-03

**Authors:** Zakaria Ouhaz, Brook A. L. Perry, Kouichi Nakamura, Anna S. Mitchell

**Affiliations:** 1 Department of Experimental Psychology, University of Oxford, Oxford OX1 3SR, United Kingdom; 2 Institut Supérieur des Professions Infirmières et Techniques de la Santé, Marrakech 40000, Morocco; 3 Medical Research Council Brain Network Dynamics Unit, Nuffield Department of Clinical Neurosciences, University of Oxford, Oxford OX1 3TH, United Kingdom

**Keywords:** anterior cingulate, cognitive control, cognitive flexibility, decision making, medial prefrontal cortex, orbitofrontal cortex

## Abstract

Cognitive flexibility, attributed to frontal cortex, is vital for navigating the complexities of everyday life. The mediodorsal thalamus (MD), interconnected to frontal cortex, may influence cognitive flexibility. Here, male rats performed an attentional set-shifting task measuring intradimensional (ID) and extradimensional (ED) shifts in sensory discriminations. MD lesion rats needed more trials to learn the rewarded sensory dimension. However, once the choice response strategy was established, learning further two-choice discriminations in the same sensory dimension, and reversals of the reward contingencies in the same dimension, were unimpaired. Critically though, MD lesion rats were impaired during the ED shift, when they must rapidly update the optimal choice response strategy. Behavioral analyses showed MD lesion rats had significantly reduced correct within-trial second choice responses. This evidence shows that transfer of information via the MD is critical when rapid within-trial updates in established choice response strategies are required after a rule change.

## Significance Statement

We demonstrate for the first time that rodent mediodorsal thalamus (MD) is a critical node when choice response strategies need to change rapidly after a within-session rule change during reward-guided learning. MD interactions with orbitofrontal cortex (OFC) are critical for value-based learning, while MD interactions with medial prefrontal cortex (PFC) are critical for rapid within-trial updating of optimal choice response rules. MD interactions with the OFC are not always necessary for reversal learning. These results indicate that the MD also contributes to attentional set-shifting. Our evidence advocates for studies investigating deficits in updating optimal choice response strategies because of potentially disrupted cortico-thalamocortical transfer of information in people diagnosed with Alzheimer’s disease (AD) combined with more frontal pathology, Parkinson’s disease (PD), or schizophrenia.

## Introduction

Cognitive flexibility describes our ability to quickly and selectively switch our thoughts, responses, or behavior to everyday dynamic situations. This capacity to rapidly update or alter one’s actions conveys evolutionary benefit and is key to survival (Koechlin et al., 2003; Rougier et al., 2005). Cognitive flexibility shows marked changes or declines in neurodegenerative disorders, like Parkinson’s disease (PD), in patients with ventral prefrontal cortex (PFC) damage, and in neurodevelopmental diseases, like schizophrenia (Clark et al., 2004; Andrews et al., 2006; Barch et al., 2009; Giraldo-Chica et al., 2018).

Cognitive flexibility is not unique to primates. Studies show that rats can readily switch between attentional sets to optimize reward outcome (Birrell and Brown, 2000; McAlonan and Brown, 2003; Newman and McGaughy, 2011). Typically, these studies have focused on various frontal cortex subregions. Yet, it is becoming increasingly clear that the cortex does not function in isolation, but rather, relies heavily on subcortical and peripheral inputs supplied by the thalamus (Sherman and Guillery, 2002; Guillery and Sherman, 2011; Halassa and Kastner, 2017). The mediodorsal thalamus (MD) has a critical role in functions of frontal cortex during higher order cognitive processes across mammalian species (Mitchell and Dalrymple-Alford, 2005; Mitchell et al., 2007a, b; Mitchell and Gaffan, 2008; Parnaudeau et al., 2013; Browning et al., 2015; Chakraborty et al., 2016, 2019; Miller et al., 2017; Schmitt et al., 2017; Alcaraz et al., 2018; Ferguson and Gao, 2018; Pergola et al., 2018; DeNicola et al., 2020; Perry et al., 2021). This evidence highlights that the MD and PFC are working in partnership, contributing different but complementary roles to goal-directed behaviors (Mitchell, 2015; Perry et al., 2021).

In mammals, the MD is densely interconnected with areas of frontal cortex involved in various aspects of cognitive flexibility. Recent work shows MD projections to frontal cortex allow for the simultaneous sharing of information across multiple cortical regions. This distributed pattern of frontal innervation by the MD appears to be especially true for the central (MDc) and medial (MDm) subdivisions, which have projections to the orbitofrontal cortex (OFC), medial PFC, and frontal association areas (Groenewegen, 1988; Dajani and Uddin, 2015; Alcaraz et al., 2016; Kuramoto et al., 2017; Perry et al., 2021). Although phylogenetic differences exist in the organization of rodent and primate frontal cortex (Preuss, 1995), similar functional contributions to aspects of cognitive flexibility are observed in monkeys and rodents after perturbations to similar areas of agranular frontal cortex (Dias et al., 1996, 1997; Birrell and Brown, 2000; McAlonan and Brown, 2003; Bissonette et al., 2008). Given there are similarities in neuroanatomical connectivity between agranular frontal cortex and MD in rodents and primates, it seems that cognitive flexibility and rapid updating deficits observed in monkeys with excitotoxic MD lesions (Mitchell et al., 2007a; Mitchell and Gaffan, 2008; Browning et al., 2015; Chakraborty et al., 2016) may also extend to rodents.

Thus, in the current study, rats with excitotoxic lesions to the MD or MD sham controls were run on a well-established test of cognitive flexibility, the intradimensional (ID)/extradimensional (ED) attentional set-shifting task (Birrell and Brown, 2000). To test this premise and investigate any deficits in choice responding, we used the standard 7-stage version comprising of multiple subtasks all conducted within a single testing session (Birrell and Brown, 2000; Chase et al., 2012). In rats, the ID/ED task has produced dissociable deficits after permanent cortical or limbic thalamic lesions, or neuropharmacological manipulations (McGaughy et al., 2008; Tait et al., 2014, 2018; Wright et al., 2015; Linley et al., 2016).

In the current experiment, we predicted MD lesion rats would show deficits during the initial sensory acquisition (simple discrimination; SD) and the ED shift. Both the SD and ED subtasks require rapid, within trial learning or updating of choice response strategies. Therefore, impaired SD and ED performance would be consistent with the selective learning deficits observed in other rodent studies after MD perturbations (Mitchell and Chakraborty, 2013; for review, see Hunt and Aggleton, 1998; Mitchell and Dalrymple-Alford, 2005; Ferguson and Gao, 2018; Courtiol et al., 2019; Perry et al., 2021). In addition, we predicted our MD lesion rats may show deficits during reversal subtasks. MD lesions in rodents can produce reversal learning deficits, although the evidence is mixed (for review, see Mitchell and Chakraborty, 2013). However, given the reciprocal direct MD connectivity with the OFC (Krettek and Price, 1977; Groenewegen, 1988; Ray and Price, 1992), and the selective deficits observed in rodents and monkeys with OFC manipulations performing the reversal subtasks during the ID/ED attentional set-shifting task (Dias et al., 1996; McAlonan and Brown, 2003), it remained an open question.

## Materials and Methods

### Animals

Twenty-five Lister hooded male rats weighing between 420 and 480 g at time of surgery (Charles River) were group housed in a temperature and humidity-controlled environment (21 ± 1°C). The housing and husbandry compiled with the ARRIVE guidelines of the European Directive (2010/63/EU) for the care and use of laboratory animals. Testing was conducted in the light phase of a 12/12 h light/dark cycle (lights on at 7 A.M.s). The rats were maintained on a controlled feeding schedule (20 g/rat/d) with water freely available in the home cage. All experimental procedures were performed in compliance with the United Kingdom Animals (Scientific Procedures) Act of 1986. A project license authorized all procedures after review by the university animal care and ethical review committee and Home Office Inspectorate.

### Surgery

Rats were anesthetized by isoflurane and oxygen mix (4% induction, 1.8–2% maintenance), and given analgesia in subcutaneous injections of 0.05 mg/kg buprenorphine (Vetergesic; 0.3 mg/ml) and 1 mg/kg Metacam (Meloxicam; 5 mg/ml). Rats were secured in a stereotaxic frame (Kopf) with atraumatic ear bars and the nose bar set to +3.3 mm to achieve a level skull. A subcutaneous injection of 2 mg/kg bupivacaine (Marcaine; 2.5 mg/ml) was administered into the scalp in the location of the midline incision. Viscotears was applied to keep the eyes moist. During surgery, each rat was placed on a heat pad and covered in bubble wrap with an internal rectal thermometer probe to monitor and maintain normal body temperature. Warmed sterile saline (1 ml/100 g) was administered subcutaneous into the scruff of the neck after 1 h. A midline incision was performed. Bregma and λ coordinates were determined. A dental drill with a trephine head was used to create a craniotomy over the midline. To maximize lesion accuracy, anterior and posterior injection sites were calculated according to the bregma–λ distance in each rat. Coordinates were for anterior MD injection: anterior-posterior (AP) = – 0.395 mm, medial-lateral (ML) = – 0.1 (avoiding the superior sagittal sinus, which was visible within the dura inside the craniotomy), and dorsal-ventral (DV) = 0.56 mm (from dura), volume of excitotoxin = 0.20 μl; for posterior MD: AP = – 0.435 mm, ML = + 0.1 and DV = 0.57 mm (from dura), volume of excitotoxin = 0.18 μl. Fifteen rats (MD lesion) received 0.12 m NMDA dissolved in phosphate buffer (pH 7.20) in each hemisphere from a 1 μl-gauge Hamilton bevelled-tip syringe. The injections were performed manually, taking 3 min each, and after injection the needle was left in situ for a further 3 min to allow diffusion and to limit wicking of the NMDA. A further 10 rats (MD sham controls) received injections of sterile phosphate buffer instead of NMDA using the same injection coordinates. Injections were given bilaterally in the MD. Upon completion of surgery, wounds were sutured with Vicryl 4.0. Rats were housed singly during recovery for up to 3 d and were then returned to their presurgery housing cohort. Postoperative oral analgesia, 1 mg/kg Metacam (Meloxicam, 1.5 mg/ml) dissolved and set in jelly was provided in individual bowls to each rat daily for 3 d. Behavioral and physiological evidence showed that all rats recovered well, with normal eating and presurgery weights returning within 24 h. Food regulation (20 g/d/rat) started again 10d after recovery. Postoperative testing began at least 15d after surgery. The researcher was blind to the lesion group of each animal until all tests were completed.

### ID/ED attentional set-shifting paradigm

#### Apparatus

The test chamber consisted of a modified Plexiglas home cage (40 × 70 × 18 cm) with Perpex dividers separating the cage into a large start chamber (40 × 46 cm) and two identically sized (24 × 20 cm) choice chambers to which access was controlled by removable Perspex doors. The digging bowls were ceramic (internal diameter 7 cm, depth 4 cm) and were placed within the choice chambers. The bowls were filled with digging media of different textures, and the digging media were scented with different spices (see Table 1 for examples). The odor or digging media discriminations, pairs of stimuli used, and the correct stimulus within pairs were counter-balanced across subtasks and matched between groups. The bowls were baited with Honey Nut Cheerios (Nestle), each bowl contained a hidden (securely fixed under a metal grid) Honey Nut Cheerio to serve as an odor mask. During testing, only one of the bowls was baited and rats determined which bowl was baited using either the texture of the digging medium or the odor as cues. Before testing, rats were taught to dig in bowls filled with home-cage bedding material to retrieve one half of a Honey Nut Cheerio. The task was performed as previously described in Birrell and Brown (2000) and divided into two phases administered on two consecutive days.

**Table 1.** Sensory dimensions (odors and digging media) and stimulus features (O1–O6; M1–M6) used in the different subtasks of the ID/ED attentional set-shifting task

Odors		Digging media	
O1, cinnamon	O2, ginger	M1, coarse tea	M2, fine tea
O3, sage	O4, paprika	M3, sand	M4, grit
O5, turmeric	O6, cloves	M5, coarse shavings	M6, fine shavings

#### Preoperative and postoperative training day

On the day before testing, each rat learnt one simple two-choice discrimination (SD) using either of the two sensory dimensions: odor (mint vs oregano), or digging media (shredded paper vs cotton pads), to a criterion of six consecutive correct trials. The rewarded odors or digging media were counterbalanced across the two groups, and these exemplars were not used again throughout testing. Digging was defined as active digging with both front paws or active foraging with the snout in the digging media. Sniffing or touching the media with the paws was not scored as a dig.

### Preoperative and postoperative testing paradigm

The following day, each rat was given a series of seven two-choice discriminations (subtasks) in the following order: a new SD using either of two sensory dimension (odor or digging media); a compound discrimination (CD) using the same rewarded sensory dimension and two-choice discrimination as the SD, combined with the other irrelevant sensory dimension; first reversal (REV1), in which the exemplars remained the same as in the CD but the correct (rewarded) and incorrect exemplars were reversed; ID shift, in which new exemplars were used, but the relevant sensory dimension remained the same as in the previous subtasks; second reversal (REV2), where exemplars in the ID remained the same but the reward contingencies of the two exemplars were reversed; ED shift, where new exemplars were used and the previously irrelevant sensory dimension becomes relevant; and a third reversal (REV3), where exemplars in the ED subtask remained the same but the reward contingencies of the two exemplars were reversed.

The task is self-paced and relies on the natural foraging behaviors of the rats. The first five two-choice discrimination subtasks (SD, CD, REV1, ID, REV2) must be solved by discriminating between exemplars from the same sensory dimension. In this stage of the task, the rat is rewarded for choices based on specific perceptual features of the stimuli, while ignoring other features that also distinguish the stimuli. After acquiring each set of two-choice discriminations to a performance criterion of six correct consecutive trials, the rats encounter a reversal of the reward contingencies associated with the exemplars (a reversal subtask: REV1 and REV2). For the final two subtasks (ED and REV3), exemplars from the previously irrelevant sensory dimension become relevant. Therefore, the rat must shift its attention (and adjust its choice response strategy) to the previously irrelevant sensory dimension and perceptual feature to receive reward (ED subtask). The attentional set-shifting cost is calculated by comparing trials to criterion during the ID shift, where the sensory dimension is consistent with previous subtasks, and the ED shift, where the now relevant sensory dimension had been previously ignored and thus requires a shift to optimize continuing to retrieve rewards.

### Odor detection test

At the completion of ID/ED testing, the olfactory abilities of the rat to smell the odor of the buried half-Honey Nut Cheerio were assessed. This task consisted of 10 consecutive trials where the rat was exposed to bowls containing similar bedding. The rat was placed in the waiting area of the set-shifting apparatus. Similar bedding-filled bowls were placed, one in each of the choice chambers, only one bowl contained half a Honey Nut Cheerio at the bottom (pseudorandomly assigned to avoid the rat developing a response strategy). The barrier was raised, allowing the rat access to both bowls. The rat had to chose to dig into one bowl and had up to 15 min to make a digging response on each trial. If the rat dug in the correct bowl, the trial was recorded as correct and the rat was returned to the waiting area and the barrier lowered to block access to both choice chambers. If the rat dug in the incorrect bowl, the trial was marked as incorrect. The rat was permitted to continue to explore the incorrect bowl; the trial was terminated when the rat returned to the waiting area. In all cases, the ability of each rat to detect the reward location was no different to chance guessing.

### Perfusion and histology

At the end of all experimental testing, rats were deeply anaesthetized with sodium pentobarbital (Euthanol 1.0 ml/rat, 200 mg/ml: Merial), and perfused transcardially with 0.9% (w/v) saline and 4% (w/v) paraformaldehyde in 0.1 m PBS. Brains were then removed and postfixed overnight (4°C), then incubated using 30% (w/v) sucrose in 0.1 m PBS over 48 h (4°C). Free floating sections (40 μm) were cut on a freezing microtome, the slices were then preserved in cryoprotectant (0.1 m PBS containing 24% (v/v) glycerol and 24% (v/v) ethylene glycol) and stored at −20°C until required. MD thalamus lesion locations were histologically verified from cresyl violet-stained brain sections.

### Lesion extent

Photomicrographs of the Nissl-stained MD sections were captured using a camera mounted on a freestanding Leica DMR microscope (Leica Microsystems) using a 1.6× objective so that the whole section could be visualised in the photomicrograph. MD volumes were measured using the NIH software ImageJ (http://rsbweb.nih.gov/ij/). In each rat, the intact MD volume from both the left and the right hemisphere was assessed. The final reading was calculated according to the Cavalieri principle (Regeur and Pakkenberg, 1989) and expressed as the percentage of MD sham controls.

### Behavioral data analysis

In the behavioral experiments, all rats performed a preoperative and postoperative session, and they completed the seven two-choice discrimination subtasks in an identical order. Data are expressed as mean and standard deviation. Preoperative and postoperative mean trials to criterion, shift cost, percent correct second choice within trial responses, and latency to dig (seconds) were analyzed using repeated measures ANOVAs with Stage (comprising the seven subtasks) as the within-group repeated measure and Lesion_type (MD lesion or MD sham control) as the between-group factor. For significant interactions, *post hoc* simple main effects analyses were performed using additional two-way ANOVAs for repeated measures (e.g., ID/ED shift) or independent *t* tests for relevant subtasks (corrected for multiple comparisons). Univariate ANOVA was used to determine whether the Shift between the two sensory dimensions between the MD lesion or MD sham control had an effect on the number of errors performed in the ED subtask. All statistical analyses were calculated using SPSS 24 software. The significance level was set at *p* < 0.05.

## Results

### MD lesion site

**Figure 1.**
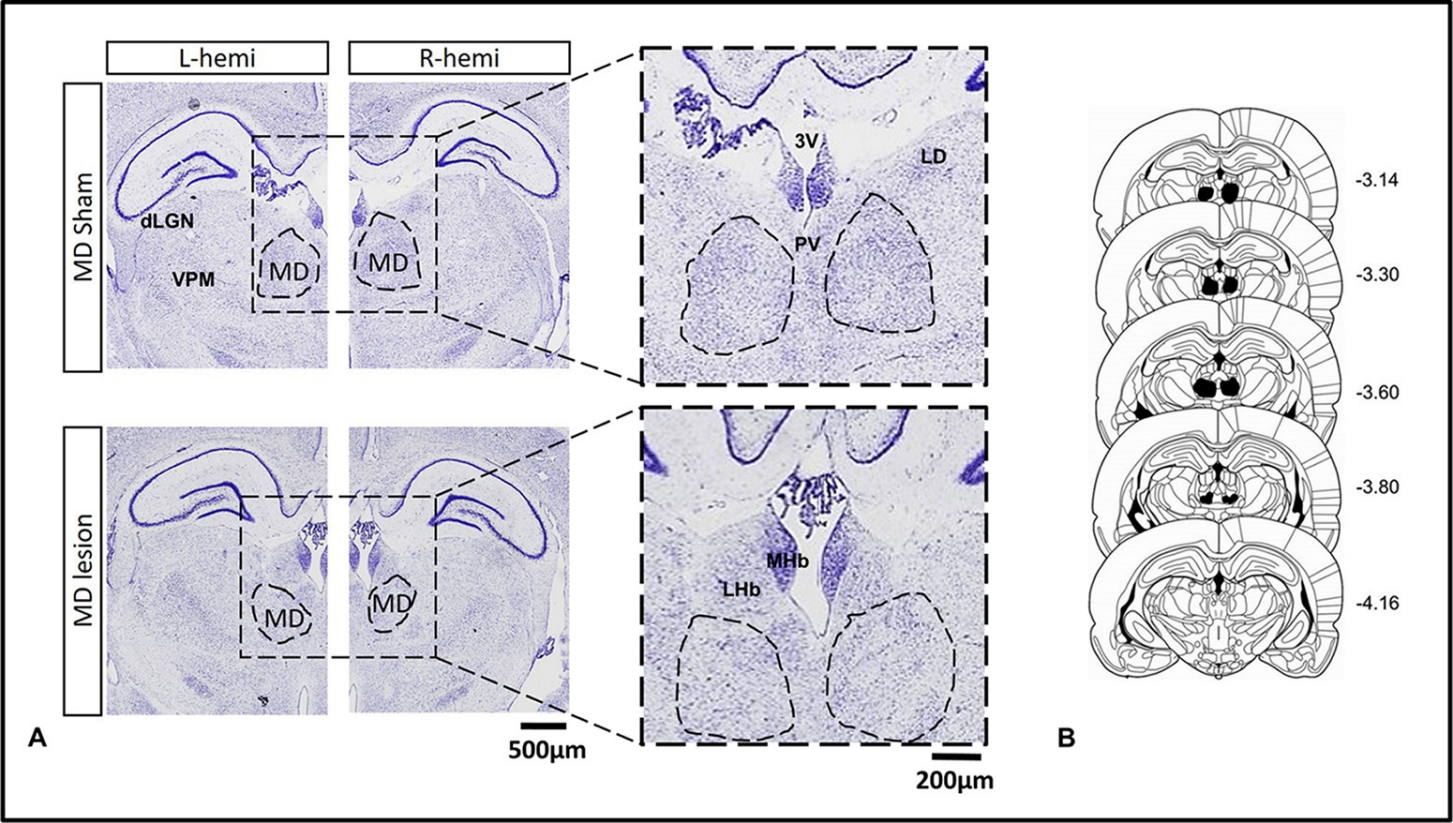
***A***, Photomicrographs of the thalamus in a MD sham control rat (top) and a MD lesion rat (bottom). ***B***, A series of coronal schematics throughout the MD showing the area of cell loss in the MD lesion group. Numbers refer to the distance from bregma (Paxinos and Watson, 2008). 3V: third ventricle; dLGN: dorsal lateral geniculate nucleus; LHb: lateral habenula; LD: laterodorsal thalamus; MD: mediodorsal thalamus; MHb: medial habenula; PV: paraventricular nucleus; VPM: ventral posterior medial thalamus.

Of the 25 adult rats involved in this experiment, 15 sustained MD lesions and 10 were MD sham controls (for details, see Materials and Methods). All 15 MD lesion rats had extensive excitotoxic (NMDA) lesion damage (Fig. 1) within the medial and central subdivisions of the MD, as intended. In all cases the lesion affected between 80% and 90% of the central and medial MD, sparing only the lateral portions. The MD was consistently shrunken, and at the lesion site there was significant cell loss. In all cases, the adjacent anterior thalamic nuclei (ATN) were spared. In two cases, there was unilateral (right-sided) damage in the lateral habenulae (LHb) and some damage to the central medial nucleus lying underneath the MD.

### The MD and attentional set-shifting

**Figure 2.**
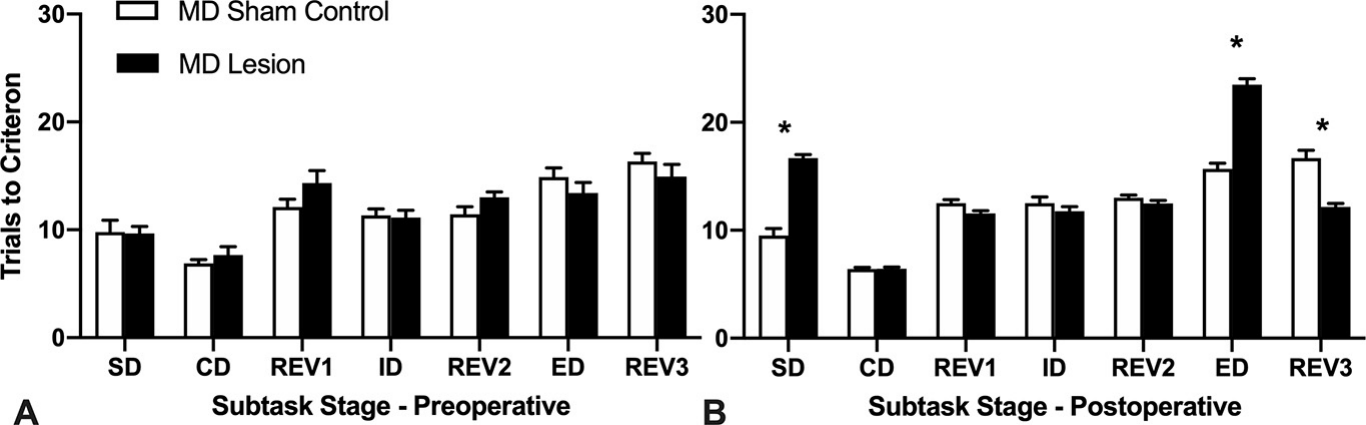
Mean (+SEM) trials to criterion data for the attentional set-shifting task. ***A***, Preoperative test session. ***B***, Postoperative test session. MD lesion rats were significantly slower to learn the SD and the ED subtasks compared with MD sham controls (*p < 0.001). MD sham control rats, like MD lesion rats, needed more trials to learn the ED, as expected. However, the MD lesion rats required significantly fewer trials to criterion to learn REV3, the reversal occurring immediately after the ED, compared with the MD sham controls (*p < 0.001). Also see Extended Data Figure 2-1 showing boxplots of the mean trials to criterion for individual rats during the postoperative session of the attentional set-shifting task.

Preoperatively, rats were pseudo-randomly assigned to either of two groups: MD lesion or MD sham control, based on the number of trials needed to reach the learning criterion (six correct consecutive trials in nine trials) during the SD subtask. Preoperative data from one MD sham control rat had to be discarded. An independent samples *t* test confirmed there was no significant difference between the two groups during preoperative learning of the SD, *t*_(22)_ = 0.095, *p *>* *0.05, with rats from both groups requiring a similar number of trials to reach criterion, MD lesion (mean (M) = 9.67, SD = 2.44) and MD sham control (mean = 9.78, SD = 3.31). Overall trials to criterion during the preoperative training session are presented in Figure 2*A*.

10.1523/ENEURO.0162-21.2022.f2-1 Extended Data Figure 2-1 Boxplots of the mean trials to criterion for individual rats during the postoperative session of the attentional set-shifting task. MD lesion rats took significantly more trials to learn the SD and the ED subtasks compared to MD sham controls (*p < 0.001). MD sham control rats also needed more trials to learn the ED, as expected. The MD lesion rats required significantly fewer trials to learn REV3, the reversal occurring immediately after the ED, compared to the MD sham controls (*p < 0.001). Box shows 1st and 3rd quartile and whiskers are the minimum and maximum trials to criterion for individual rats. Download Figure 2-1, TIF file.

Postoperatively, a repeated measures ANOVA with Stage (7 subtasks: SD, CD, REV1 ID, REV2, ED, REV3) as the within-group factor and Lesion_type (MD lesion vs MD sham control) as the between-group factor revealed a significant interaction, Stage × Lesion_type, *F*_(6,138)_ = 63.65, *p* < 0.001, a significant main effect of Stage, *F*_(6,138)_ = 184.73, *p* < 0.001, and a significant main effect of Lesion_type, *F*_(1,23)_ = 20.22, *p* < 0.001 (Fig. 2*B*). To explore the interaction effect, *post hoc* comparisons of the simple main effects of Stage (corrected for multiple comparisons, *p* < 0.007; α = 0.05 divided by seven tests) were computed. For the SD subtask, MD lesion rats required more trials to criterion (M = 16.67, SD = 1.23) compared with MD sham controls (M = 9.50, SD = 2.07), indicating that the rats with the MD lesion were slower to acquire the new sensory discrimination. An independent samples *t* test confirmed this difference was significant, *t*_(23)_ = 10.88, *p* < 0.001. However, once the rats with MD lesion acquired the rewarded sensory dimension (to respond either to the digging media, or to the odor), they were unimpaired in subsequent subtasks that maintained the same rewarded sensory dimension, namely, concurrent discrimination (CD): *t*_(23)_ = 0.00, *p* =1.0, and ID shift: *t*_(23)_ = 1.08, *p *<* *0.291. MD lesion rats were also not impaired in the reversals (REV1 and REV2) of the reward contingencies associated with the learned exemplars completed after the CD subtask, REV1: *t*_(23)_ = 2.31, *p *<* *0.030, or after the ID subtask, REV2: *t*_(23)_ = 1.34, *p *<* *0.192, although, all rats were familiar with the reversal rule as they had completed the ID/ED task preoperatively (see Materials and Methods). Extended Data Figure 2-1 shows the mean and first and third quartile box plots with the smallest and largest numbers of postoperative trials to criterion (whiskers) for the individual rats during each of the subtasks.

For the ED subtask, the rats had to shift their attention to the previously irrelevant (unrewarded) sensory dimension. Thus, given the rats had acquired the attentional set strategy, as expected, all rats required more trials to criterion to learn the ED subtask. MD sham controls required (M = 15.70, SD = 1.64) more trials to learn the ED when compared with trials required to learn the ID (M = 12.5, SD = 1.84). Similarly, the MD lesion rats required more trials (M= 23.47, SD = 2.07) compared with trials to learn the ID (M = 11.73, SD = 1.67; see Fig. 2*B*). A repeated measures ANOVA computing the trials to criterion for these two subtasks (ID vs ED) showed a significant Stage × Lesion_type interaction, *F*_(1,23)_ = 99.96, *p* < 0.001, a significant effect of Stage, *F*_(1,23)_ = 306.11, *p* < 0.001, and a significant effect of Lesion_type, *F*_(1,23)_ = 32.83, *p* < 0.001 (Fig. 2*B*). The interaction occurred as the MD lesion rats required significantly more trials to criterion to consistently implement a new choice strategy to learn which of two stimuli was rewarded from the previously ignored sensory dimension compared with the MD sham controls, *t*_(23)_ = 10.00, *p* < 0.001.

Interestingly, for REV3, the reversal subtask that occurred after the ED shift, MD lesion rats required fewer trials to criterion (M = 12.13, SD = 1.25) compared with the MD sham controls (M = 16.7, SD = 2.26; see Fig. 2*B*). An independent samples *t* test confirmed this difference was significant, *t*_(23)_ = 6.51, *p* < 0.001, suggesting a facilitation in performance as a consequence of over-training experienced during the ED subtask (see Discussion for interpretation).

**Figure 3.**
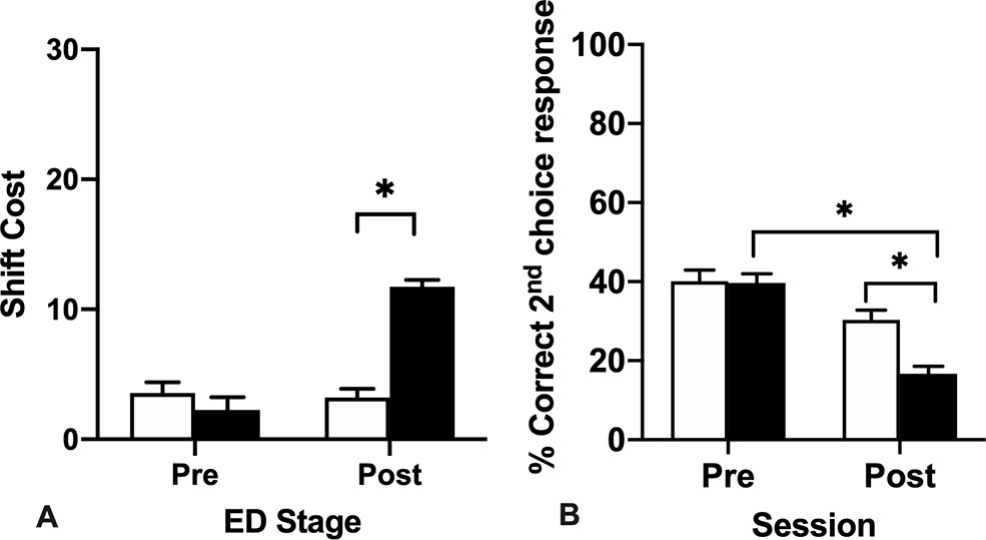
***A***, Mean (+SEM) trials to criterion shift cost data for the ED stage for preoperative (Pre) and postoperative (Post) testing sessions. The shift cost is computed as the number of trials to criterion in the ED minus the number of trials to criterion in the ID. Both groups of rats showed a shift cost during the ED, when they had to learn to attend to the previously irrelevant dimension. However, the shift cost for the MD lesion rats in the postoperative test session was significantly different to the MD sham controls. ***B***, Mean (+SEM) percent correct second choice within trial responses made during the preoperative and postoperative performance sessions of the attentional set-shifting task. MD lesion rats made fewer correct second choice within trial responses than MD sham controls (*ps = 0.001).

The additional repeated measures ANOVA of session (preoperative vs postoperative) shift cost (Fig. 3*A*) between Lesion_type computed as the number of trials to criterion for the ED subtask minus the number of trials to criterion for the ID subtask for each session showed a significant Session × Lesion_type interaction, *F*_(1,22)_ = 44.06, *p* < 0.001, a significant effect of Session, *F*_(1,22)_ = 38.27, *p* < 0.001, and a significant effect of Lesion_type, *F*_(1,22)_ = 15.46, *p* =0.001 (Fig. 3*A*). The significant interaction occurred as there was a small shift cost for both groups during the preoperative test session. However, during the postoperative test session only the MD lesion rats showed a significant shift cost as a consequence of the task demands changing that required an adjustment to the previously well-established response strategy (*p* < 0.001). By contrast, the MD sham controls showed a similar shift cost to their preoperative performance.

### Within trial choice responses as a measure of rapid adaptability after MD lesion

Given the observed deficits in the MD lesion group, we investigated the behavioral responses made in the MD lesion or MD sham control rats. Given the task design and a criterion of six consecutive correct responses before switching to the next stage, overall during testing, the rats do not complete many trials. Nevertheless, we could determine whether the rats rapidly adapted their choice responses in the here and now (within trial choice responses) as measured by the number of correct second choice within trial responses made, i.e., when the rat dug from the correct bowl only after visiting the incorrect bowl first but without digging in it, divided by the total number of correct responses (correct first and second choice responses combined) for each session. During each subtask of the session, we recorded whether the rat made a correct choice, either on the first attempt (“first choice response”), or on the second attempt (“second choice within trial response”). We did not include error trials in this analysis.

A repeated measures ANOVA of total percent correct second choice within trial responses was conducted with session (preoperative vs postoperative) as the repeated measure × Lesion_type revealed a significant interaction of Session × Lesion_type, *F*_(1,21)_ = 9.40, *p *=* *0.006, a significant main effect of Session, *F*_(1,21)_ = 57.06, *p* < 0.001, and a significant main effect of Lesion_type, *F*_(1,21)_ = 7.18, *p *=* *0.014 (Fig. 3*B*). To investigate the interaction effect, *post hoc* comparisons of the simple main effects showed that the MD lesion rats (M = 28.18, SD = 7.61) made fewer correct second choice within trial responses compared with the MD sham controls (M = 35.23, SD = 8.46). This deficit suggests the MD lesion rats had a diminished ability to rapidly bind together their previous choice about what stimuli are worth sampling to update their choice response strategy within the trial, rather than a deficit in perseverative responding, or an inability to respond to negative feedback.

In addition, we analyzed the rats’ errors made reaching criterion during the ED subtask, as a consequence of whether they experienced a sensory dimension shift from odor to digging medium or digging medium to odor, which indicated that the increase in errors occurs regardless of the direction of the attentional shift. The univariate ANOVA with Lesion_type and Sensory Shift as the between-subject factors and errors to criterion for the ED subtask as the dependent measure revealed a main effect of Lesion_type, *F*_(1,21)_ = 106.03, *p *<* *0.001, but no main effect of Sensory Shift, *F*_(1,21)_ = 3.84, *p* = 0.063 and no interaction effect, *F*_(1,21)_ = 0.19, *p *=* *0.666.

### Latency to dig changes

**Figure 4.**
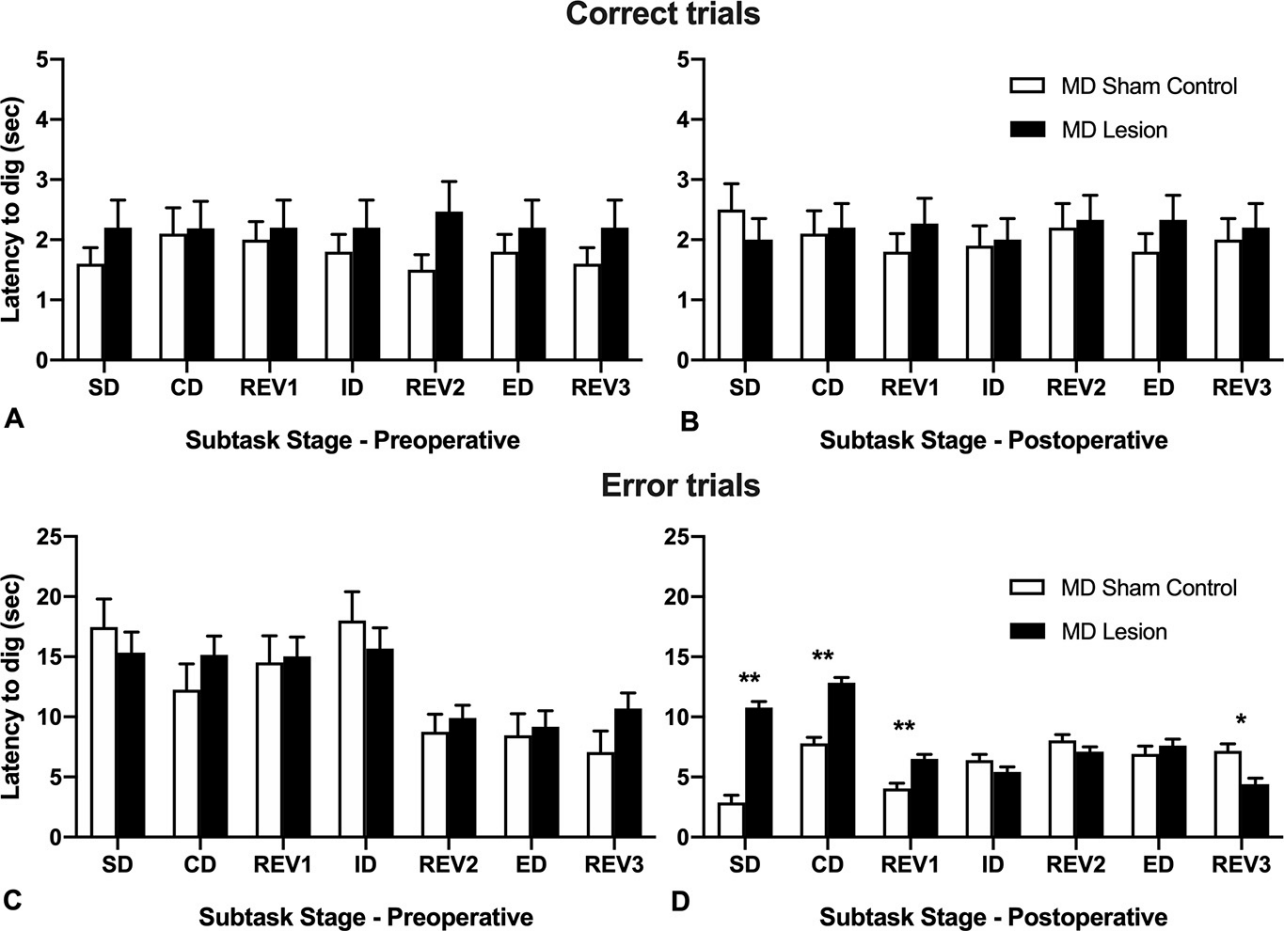
Mean (+SEM) latencies to dig (seconds) for MD sham controls and MD lesion rats during each subtask of the ID/ED attentional set-shifting task. ***A***, Preoperative correct trials. ***B***, Postoperative correct trials. ***C***, Preoperative error trials. ***D***, Postoperative error trials (**p <* *0.001, *p =* *0.002).

Latency to dig (in seconds) for error trials and correct trials were also computed for each rat in preoperative (Fig. 4*A*,*C*) and postoperative (Fig. 4*B*,*D*) analyses. Separate repeated measures ANOVAs were used to assess the latency to dig for the preoperative or postoperative sessions for either error trials or correct trials with Stage (seven subtasks: SD, CD, REV1 ID, REV2, ED, REV3) as the within-group factor and Lesion_type (MD lesion vs MD sham control) as the between-group factor.

For error trials (Fig. 4*C*), the repeated measures ANOVA for the preoperative session revealed a significant main effect of Stage, *F*_(1,21)_ = 23.99, *p* < 0.001, but no effect of lesion, and no interaction, *F*s < 1.0. This result indicates there was no difference in response times between the pseudo-randomly assigned groups (MD lesion and MD sham controls) preoperatively and that all rats responded quicker on errors trials as they moved through subsequent subtasks within the session.

In contrast, the repeated measures ANOVA of latency to dig during postoperative error trials (Fig. 4*D*) revealed a significant interaction of Stage × Lesion_type, *F*_(6,132)_ = 26.81, *p* < 0.001, a significant main effect of Stage, *F*_(6,132)_ = 22.45, *p* < 0.001, and a significant main effect of Lesion_type, *F*_(1,22)_ = 57.15, *p* < 0.001. To investigate the interaction effect, *post hoc* comparisons of the simple main effects (corrected for multiple comparisons) were computed. Postoperatively, MD lesion rats responded slower on error trials during the SD subtask, *t*_(22)_ = 10.09, *p* < 0.001, the CD subtask, *t*_(22)_ = 7.65, *p* < 0.001, and the REV1 subtask, *t*_(22)_ = 4.44, *p* < 0.001. However, for the final reversal subtask, REV3, completed after the ED shift, MD lesion rats were quicker on the error trials than MD sham controls, *t*_(22)_ = 3.53, *p *=* *0.002. This result in combination with the reduced trials to criterion required to complete this subtask (Fig. 2*A*) suggests that the MD lesion rats may have experienced overtraining because of the number of additional trials to criterion required to adapt their choice responding in the ED subtask (Dhawan et al., 2019). For correct trials, repeated measures ANOVAs for the preoperative or postoperative session showed no significant main effects and no significant interaction (*p*s > 0.05).

## Discussion

This set of experiments investigated the active influence of rat MD in cognitive flexibility. More specifically, we examined whether rats with excitotoxic lesions of the central and medial MD were impaired in performing an attentional set-shifting task that measures ID and ED shifts in attention to sensory discriminations (digging media or odors). Our new results indicate that rats with MD lesions required more trials to criterion to learn the optimal response strategy during the initial SD. Although, once this stable response strategy had been learnt, MD lesion rats were able to attain an attentional set strategy as they acquired further two-choice discriminations involving the same sensory dimension during the CD and ID shifts, and during reversals (REV1 and REV2) of the reward contingencies associated to the exemplars. However, when the relevant sensory dimension was switched, for the ED subtask, the MD lesion rats were markedly impaired and required many more trials to update their choices using a new response strategy, as measured by the significant ED shift cost. Further, for REV3, the third reversal performed after the ED shift, the MD lesion rats showed facilitated learning, suggesting they had received “overtraining” as a consequence of completing many more trials to reach criterion in the ED subtask (see below). The analyses of behavioral responses indicated that the MD lesion rats made significantly fewer correct second choice within-trial responses suggesting that information transfer via the MD is critical for the rapid, within-trial monitoring and updating of an optimal choice response. Interestingly, similar updating deficits in choice responding have been previously reported in rhesus macaques with magnocellular MD excitotoxic lesions (Chakraborty et al., 2016).

### Learning and updating

These behavioral data accord somewhat with previous studies conducted in different species. For example, marmosets with lateral PFC lesions (Dias et al., 1996) or rats with medial PFC lesions (equivalent frontal regions; Birrell and Brown, 2000) are also impaired at the ED subtask, showing a similar ED shift cost as our MD lesion rats. However, unlike our MD lesion rats, these marmosets and rats did not need more trials to reach criterion during the SD subtask. Instead, the MD lesion rats’ learning deficit during the SD subtask accords with rats that had ATN lesions, who similarly required more trials to acquire the SD subtask (Wright et al., 2015), although these ATN lesion rats were not impaired during the ED subtask. Instead, and in contrast to our MD lesion rats, rats with ATN lesions continued to be impaired during the CD subtask and subsequent ID shifts (Wright et al., 2015) indicating that they never properly learnt the discrimination rule, which suggests an intact ATN (a brain structure adjacent to the MD but with markedly different cortico-thalamo-cortical connectivity) is important for supporting the formation of an attentional set strategy.

As already indicated, with more trials, our rats with MD lesions were able to learn the SD subtask to criterion and apply the optimal rule during subsequent subtasks. For the SD subtask, trials revert back to presenting only one sensory dimension and two stimulus exemplars. Other studies also confirm MD lesion rats are impaired in acquisition of an initial task rule (Hunt and Aggleton, 1998). Other rodent studies causing temporary perturbations to the MD also show impaired discrimination learning (Ferguson and Gao, 2018; Courtiol et al., 2019). Thus, these results from across species reinforce the notion that the MD is not simply mimicking the PFC (Mitchell et al., 2007a, 2014; Mitchell and Gaffan, 2008; Miller et al., 2017; DeNicola et al., 2020). Consequently the influence of other corticocortical interactions that also indirectly transfer information to the cortex via the MD and other thalamic structures, e.g., those PFC interactions with limbic structures in the temporal lobes and sensory association areas are also critical during learning sensory discriminations (Floresco and Grace, 2003; Mitchell, 2015; Bueno-Junior and Leite, 2018; Chakraborty et al., 2019; Banerjee et al., 2020; Pelekanos et al., 2020).

However, during the ED subtask, our MD lesion rats were markedly impaired at rapidly adapting this now well-established choice response strategy when they had to attend to the previously irrelevant (nonrewarded) sensory dimension. The demands on cognitive flexibility, response inhibition, and adapting behavioral responses after negative feedback are increased when the animals must make an ED shift (Dhawan et al., 2019). The increased effort involved in adapting to these changes in attention is measured by a shift cost, and it is expected that more trials to criterion are required during learning the ED than during learning the ID shift. While the ID/ED shift cost was higher for both groups (Fig. 2*B*), it was significantly increased for the MD lesion rats compared with the MD sham controls. As indicated, increased shift costs have also been observed in marmosets and rats with lesions to the comparative PFC regions (Dias et al., 1996; Birrell and Brown, 2000). Surprisingly though, rats with damage to the nucleus reuniens (Re), another thalamic structure located near the MD, which interconnects the medial PFC and hippocampus (Hoover and Vertes, 2007, 2011; Vertes et al., 2015) do not cause deficits in the ED subtask or produce a significant ED shift cost. Instead, rats with NRe damage are impaired at acquiring the attentional set strategy, similar to rats with ATN lesions (Linley et al., 2016). Thus, our MD thalamus results are unique.

Evidence from studies in mice suggests the MD supports the frontal cortex to sustain intracortical attentional control without transferring categorical information about a particular task rule (Schmitt et al., 2017), or that medial PFC-MD projections are important for behavioral flexibility but not task engagement (Marton et al., 2018; Nakayama et al., 2018). Further, the MD has been shown to have a critical role in maintaining the balance between excitation and inhibition in dorsomedial PFC via its influence on interneurons (Ferguson and Gao, 2018; Anastasiades et al., 2021) and pyramidal neurons (Delevich et al., 2015; Collins et al., 2018). Our results are supportive of these circuit level interactions, although our deficits indicate the influence of MD on frontal circuits is specific to certain aspects of the attentional set-shifting task, e.g., linked to the ED shift and learning the optimal choice response strategy, but not to the ID shift or completing reversals. However, our study involved permanent MD lesions, which are likely to have caused some adaptations across the network, while the studies in mice likely involve short-term changes as temporary inactivation of the MD was employed. Nevertheless, it may be proposed that after MD perturbations, while the animal is still able to detect a change in the task demands (i.e., they are responsive to negative feedback), they are impaired at rapidly adapting their behavior and coordinating correct choice responses within a trial after a change to the already established choice response strategy (Hunt and Aggleton, 1998; Block et al., 2007; Chakraborty et al., 2016). Interestingly though, in both these rodent studies (Hunt and Aggleton, 1998; Block et al., 2007), the learning deficits were attributed to increased perseverative responding. Others have also observed increased perseverative responding after MD perturbations (Ferguson and Gao, 2018). In contrast, our current rodent work and previous work in rhesus macaques has shown an intact MDmc is necessary for rapid reward guided learning of complex discriminations (Mitchell et al., 2007a; Chakraborty et al. 2016, 2019), although the deficits linked to learning in monkeys are attributed to increased response switching (Mitchell et al., 2007a; Chakraborty et al., 2016). These congruent results across species after MD damage suggest that cortical information transfer via the MD is particularly important when rapid, within a trial changes in choice response strategies linked to establishing a new rule are required.

### Reversal learning

For the reversal subtasks (REV1 and REV2), somewhat surprisingly, our MD lesion rats were unimpaired during reversals in the reward contingencies. Rats and marmosets with comparable medial PFC damage are also not impaired in reversal learning subtasks (Dias et al., 1996; Birrell and Brown, 2000). Instead, OFC perturbations cause deficits in the reversal subtasks, while leaving performance intact during the SD and ED subtasks of the ID/ED task (Dias et al., 1997; McAlonan and Brown, 2003; Chase et al., 2012). These observations accord with a large body of research indicating that separate mammalian PFC subregions differentially contribute to learning, memory, and other cognitive functions (Clark et al., 2004; Rudebeck et al., 2008; for review, see Uylings et al., 2003; Dalley et al., 2004; Izquierdo et al., 2017).

MD is reciprocally connected to the OFC and medial PFC (Groenewegen, 1988; Ray and Price, 1992; Preuss, 1995). Thus, this double dissociation in deficits between these reciprocally interconnected MD-OFC and MD-medial PFC brain regions needs to be reconciled. First, it must be noted that the MD rats had experienced all of the subtasks (and thus were familiar with the concept of reversal learning and that it can appear in the task structure) during their preoperative test session, which may have reduced or eliminated the reversal learning deficit (Costa et al., 2015; Jang et al., 2015). Further, for the MD, while previous evidence of reversal learning deficits are reported for some tasks after MD perturbations (Hunt and Aggleton, 1998; Chudasama et al., 2001; Block et al., 2007; Ostlund and Balleine, 2008; Parnaudeau et al., 2013, 2015; Ferguson and Gao, 2018), the evidence is mixed (Beracochea et al., 1989; Alcaraz et al., 2018; Fresno et al., 2019). These mixed effects may be the consequence of differences in task structure and the way that the reversal is introduced. Other factors, including the extent of disruption caused to thalamic structures adjacent to the MD may also be a factor (Mitchell and Chakraborty, 2013; Wolff and Vann, 2019). Indeed, one recent study has identified that another thalamic structure nearby to the MD, the submedius thalamus, may contribute a role in reversal learning, instead of the MD (Fresno et al., 2019). Consequently, our current results and this above evidence highlights that the information transfer between OFC and MD is not always critical for reversal learning per se Taken together, it is clear that the MD is not simply mimicking the behavioral deficits observed after PFC damage, and instead shows that the MD and different subregions of PFC are supporting different aspects of cognitive flexibility. At least in monkeys, OFC-MDmc interactions are critical for adaptive, value based decision-making (Mitchell et al., 2007b; Izquierdo and Murray, 2010; Browning et al., 2015), while in rodent studies, the OFC-striatal part of the cortico-striatal-thalamic neural circuits have been implicating in reversal learning (Yin et al., 2005; Gremel and Costa, 2013; Izquierdo et al., 2017; Nakayama et al., 2018; Yang et al., 2021) or OFC-submedius thalamus circuits (Fresno et al., 2019). Other OFC-striatal-thalamic and OFC-thalamic interactions are potentially more involved in supporting reversal learning. In addition, thalamic inputs from the intralaminar thalamic nuclei and motor thalamus to the striatum contribute a selective role in inhibitory control and behavioral flexibility (Saund et al., 2017).

Intriguingly, our additional analyses used to investigate the types of behavioral deficits occurring after MD lesions showed that our rats made fewer correct second choice within-trial responses. Given that our MD lesion rats required more trials to criterion to learn the SD and ED subtasks, this change in behavioral responses suggests that without the MD thalamus, our rats could not rapidly adapt their choice responses as the trial was progressing (within-trial) after the changed task demands. However, this reduced correct second choice within-trial responding does not suggest the rats adopted perseverative responding (they would have potentially been impaired in reversal learning if they had) or were not learning about the stimulus and associated rewards. We can conclude this because, after acquiring the ED shift, the MD lesion rats required fewer trials to criterion to learn the final reversal subtask, REV3. This facilitation of learning during the final reversal subtask suggests that the MD lesion rats had experienced “overtraining” on all of the stimulus features associated with each sensory dimension as they required more trials to learn the ED subtask (Dhawan et al., 2019). It is well established that overtraining rats during discrimination learning can eliminate any reversal learning deficits (for review, see Reid, 1953; Lovejoy, 1966; Nilsson et al., 2015). As Pearce and Mackintosh (2010) indicate, until learning is fully consolidated, all stimulus features continue to be attended to and this seemed to be so for our rats, even with the MD lesion. Thus, we propose, in accord with others, that through experiencing these extra trials during the ED subtask, the MD lesion group may have developed a greater understanding of the rewarded and unrewarded stimulus dimensions, thus increasing the salience of the predictive cues and reducing/eliminating the number of factors that can led to an error. In essence, these extra trials in the ED subtask helped our MD lesion rats to slowly learn the optimal response strategy so when the final reversal was encountered, they were readily able to continue to implement this optimal response strategy for the sensory dimension while simply reversing the two stimulus exemplars (something they were already well-practiced at doing). An MD lesion does not render an animal or human densely amnesic or cause them to be insensitive to the receipt of rewards (Mitchell, 2015). Instead, we are proposing that after the loss of the MD, an animal or human is unable to rapidly learn a new choice response strategy or rapidly update a well-established optimal one.

However, in the Birrell and Brown (2000) study, rats with medial PFC lesions did not show a facilitation of learning during the final reversal subtask after showing a similar ED shift cost to our MD lesion rats. We cannot be certain why medial PFC lesion rats also did not show a facilitation of learning in the final reversal subtask, although exploring whether they experience overtraining is worthy of further investigation. What is known is that the medial PFC is proposed to contribute to learning via supporting the processing of error feedback related to adapting stimulus-action selection. For example, during instrumental learning, medial PFC lesions in rats contribute to the acquisition but not the expression of goal-directed actions (Ostlund and Balleine, 2005).

There may be other explanations for the SD and ED impairments observed in our MD lesion rats. With the ID/ED task, it is important to establish that the animals understand the relevant sensory dimension that is rewarded across the related subtasks within the session. Fortunately, in our animals, this transfer of knowledge was evident as the MD lesion rats showed reliable learning in the subtasks, CD, REV1, ID, and REV2, that required the implementation of choice responses (follow the same rule) to the same sensory dimension as the SD to receive reward. Further, both sham controls and MD lesion rats showed similar numbers of trials to criterion during the REV1 and REV2 subtask, which indicates that they did not favor one feature of the stimulus dimension more than the other or that they were insensitive to negative feedback. However, we only used male rats in this current study so the results might not transfer to female rats. Additionally, the MD is a subcortical node in the olfactory neural circuitry that also includes the OFC (Courtiol and Wilson, 2015; Veldhuizen et al., 2020). However, evidence collected from rodents with MD perturbations or humans with strokes affecting the MD indicates that changes to the MD do not impair olfactory discriminations (Tham et al., 2011; Courtiol et al., 2019). Moreover, our MD lesion rats showed similar levels of olfactory discriminations as the MD sham controls (for further details, see Materials and Methods).

Finally, the diffuse influence of the MD thalamocortical inputs to several frontal cortex structures (Mitchell, 2015; Alcaraz et al., 2016; Kuramoto et al., 2017; Wolff and Vann, 2019) supports previous findings implicating medial PFC-MD connectivity in flexible behaviors (Marton et al., 2018; Nakayama et al., 2018). Furthermore, the MD along with the OFC provides additional evidence for the contribution of these areas in supporting online, “here and now” reward-guided learning and decision-making (Wallis, 2007; Schoenbaum et al., 2011; Gardner et al., 2017, 2019). Intact OFC connectivity is essential to the animals’ ability to perform reward-guided learning in order that other interconnected neural networks, likely including corticocortical, cortico-striatal, and cortico-thalamo-cortical connectivity, can determine the optimal choice responses and implement the appropriate actions (Rushworth et al., 2011; Browning et al., 2015; Chakraborty et al., 2016; Nakayama et al., 2018; Perry et al., 2021).

The ID/ED task is analogous to the Wisconsin Card Sorting Test (WCST) in humans. In healthy humans performing the WCST during neuroimaging, the MD is activated during responding to negative feedback after the choice has been executed (Monchi et al., 2001). Unfortunately, thus far, humans with stroke damage in the MD have cognitive deficits that are clinically very poorly defined (Pergola et al., 2018). However, people diagnosed with Alzheimer’s disease (AD), PD, or schizophrenia show impaired attentional set-shifting performance (Owen et al., 1993; Monchi et al., 2004; Barch et al., 2009). Neuroimaging and postmortem studies show marked changes in the MD and/or ATN in these diseases (Hornberger et al., 2012; Pergola et al., 2015; Ouhaz et al., 2018; Perry et al., 2019). Our current evidence advocates for studies investigating cortico-thalamocortical transfer of information in people diagnosed with AD combined with more frontal pathology, or PD, or schizophrenia.

To conclude, excitotoxic damage to the rodent central and medial MD selectively increased the trials to criterion on the ED subtask, producing a shift cost. This selective performance deficit is similar to monkeys with lateral PFC inactivation (Dias et al., 1996, 1997) and rats with medial PFC lesions (Birrell and Brown, 2000). Further, this deficit after MD lesions contrasts with monkeys or rats with perturbations to the OFC, who are selectively impaired on reversal learning, but not on ED shifts (Dias et al., 1997; McAlonan and Brown, 2003; Chase et al., 2012). As evidenced, the frontal cortex is critically involved in value-based decision-making and reward-guided learning (Miller, 2000; Wallis, 2007; Rushworth et al., 2011). However, cortico-thalamocortical connections also contribute a role (Izquierdo and Murray, 2010; Mitchell et al., 2014; Browning et al., 2015; Chakraborty et al., 2016; Pelekanos et al., 2020). In rodents, the MD and the medial PFC together appear crucial for binding reward information and behavior (Corbit et al., 2003; Bradfield et al., 2013), as inhibition of dorsomedial PFC-projecting MD neurons results in rats having difficulties with tracking changes in action-outcome contingencies (Alcaraz et al., 2018). In the current study, we show that rat medial and central MD are critical for rapidly updating an optimal choice response strategy. That is, when the MD is damaged, there is a diminished ability to rapidly learn (within a trial) a choice response strategy, as well as update a well-established one as task demands change. Behaviorally, our MD lesion rats made less correct second choice within-trial responses, suggesting they could not rapidly alter their choice response strategy when they encountered the unrewarded stimuli on a given trial.

## Synthesis

Reviewing Editor: Laura Bradfield, University of Technology Sydney Decisions are customarily a result of the Reviewing Editor and the peer reviewers coming together and discussing their recommendations until a consensus is reached. When revisions are invited, a fact-based synthesis statement explaining their decision and outlining what is needed to prepare a revision will be listed below. The following reviewer(s) agreed to reveal their identity: Christoph Kellendonk, Robert Mair.

Thank you for your submission, the reviewers are largely in agreement that this is an interesting set of findings that add new knowledge to the literature. They have both included a series of points that require being addressed, as set out below. I would also add that as the study appears to only have used male mice, please could this be stated in the abstract and could there be a sentence added to the discussion stating that the results may not generalise to females.

Reviewer #1

This paper presents an interesting set of findings that MD lesions affect initial sensory discrimination (SD) and ED shifting of attention while sparing reversal learning and ID shifts of attention. These results are potentially important for understanding the role of rodent MD in cognitive flexibility. The experiments appear to be carefully conducted. The authors follow the well-established methods developed by Birrell and Brown (2000) to examine ID/ED attention shifting. MD lesions appear to be made skillfully and lesion extent analyzed quantitatively. C-Fos methods were used to assess neuronal activity in orbital and medial PFC 90 minutes following a second exposure to SD and ED training. These analyses lack necessary controls to determine whether MD lesions specifically affect the activity of these cortical areas during the behavioral tasks.

There are several issues that should be addressed in a revision of this paper.

As the authors note, Birrell and Brown found no effect of medial prefrontal lesions on initial SD. It is unclear why they predict that MD lesions should affect both SD and ED in the introduction (lines 73 and 74) while acknowledging that medial prefrontal lesions do not. This seems to be an important predicted distinction between MD and PFC function that should be addressed more directly.

The authors ascribe the decrease in trials to criterion for REV3 for the MD lesion group to effects of overtraining. This begs the question of why a similar effect not observed for REV 1 following SD overtraining here or for REV3 following ED overtraining in Birrell and Brown? Are there other potential explanations for this result?

The authors argue (paragraph beginning on line 529) that reduced correct 2nd choice responses represent a failure to adapt initial choice responses. Couldn’t this simply represent a failure to recognize a correct first choice (i.e failure to acquire the discrimination rule) leading to an incorrect response to the alternative pot?

The authors argue (paragraph beginning on line 558) that reduced c-Fos counts provide evidence that “MD-PFC interactions contribute to learning and cognitive flexibility”. It is not clear what to make of the c-Fos results. Comparable decreases were observed after a 90 m period that followed second exposure to SD and ED. However, there are no controls for nonspecific effects of MD lesions. Would comparable decreases have been observed for a group of rats given MD lesions with no behavioral training? Are decreases observed in OFC and PrL greater than in other areas of cerebral cortex? Given the lack of such basic controls, what do the c-Fos results add to this paper?

Reviewer #2

In this study the authors used excitatory lesions in the rat to study the impact of mediodorsal thalamus (MD) lesions on flexible behavior. Flexible behavior was tested in the Birrell & Brown odor-based rule shifting task that allows for assessing initial association, within perceptual dimension rule shifts (ID or reversal learning) and extradimensional rule shifts. Although the question addressed is not novel the data are important as they contradict previous findings and the discussion about these contradicting data will enhance our understanding of the role of the MD in flexible behavior. The experiments are well done. I have mostly comments about the selection and the interpretation of the data.

Figure 2: All animals completed the ID/ED task pre- and postoperatively. The authors should include both sets of data in Figure 2 so it can be seen how the animals performed pre-operatively. Also, I could not find in the method section that rats were tested pre- and postoperatively.

The authors interpret the deficit in the initial SD acquisition as that the rats have to learn a stable response strategy, which is impaired in MD lesioned rats. Once the rats have learned the strategy MD lesions have no effect on the learning of switched or reversed associations (as long as they are in the same perceptive domain). I have a problem with this interpretation because the rats have already acquired the response strategy pre-operatively. Thus, performing SD acquisition is not new for the rats. Is it possible that the lesioned rats forgot about or are not able to implement the previously learned SD-response strategy?

I don’t understand the argument of overtraining for the improved performance of MD lesioned mice in REV3. First, it takes MD lesioned mice longer to reach criterion in the ED shift condition thus doing more trials is not necessarily an overtraining. Second, if the mice were indeed overtrained, I would have expected a deficit and not an improvement in performance.

It is possible that the MD lesion animals never properly learned the rule. They may have been able to reach the criterion with a less firm understanding of the rule than the MD sham animals. In that case, they would also be quicker to learn the reversal as the rule that they would have to un-learn would not have as strong an association.

Figure 3: The rational and interpretation for the analysis of the 2nd correct choice is not clear to me. As per provided definition, the rat does a correct 2nd choice when the first bowl it visited was an incorrect bowl and the rat didn’t dig into the incorrect bowl. In this scenario if the rat doesn’t dig in the first bowl, this is for me an indication that the rat already understands the new rule based on previous trial outcomes. It is not clear to me what the authors mean by learning on the fly and why the deficit cannot be explained by an enhancement in perseverative responding or an inability to respond to negative feedback as explained in line 350. To address perseverative responding the authors should analyze the percentage of perseverative vs non-perseverative errors (e.g., as in Ferguson et al. 2018 Biol Psych).

There are alternative explanations for less correct 2nd choices. First, it possible that lesioned animals do less correct 2nd choices because they less often approached the incorrect bowl as the first bowl. Second, lesioned animals do less correct 2nd choices because they do more mistakes. By digging more often into the 1st bowl, when it is incorrect, they have less possibilities to do a 2nd correct choice.

Would it be easier for the animal to make the assessment of what is in each pot for trials where odor is the rule as opposed to texture? If odor is the dimension that is relevant, the animal might be able to smell the odor from a distance without approaching the pot and make a “2nd choice” without having to go visit both pots.

Figure 4: Reaction time seems not the appropriate term here as reaction times are usually in the range of milli-seconds. I would rather suggest to use the term “latency to dig”. Why is this measure only shown for error trials? It should be shown for both. The longer latencies to dig in the lesioned animals could be an indication of a change in motivation. This should be discussed.

Line 429: “The analysis of behavioral responses........is critical for monitoring and updating of choice response rules”. Again, I don’t see how the 2nd choice responses support this interpretation.

Figure 5-7: Even if the authors did not used unbiased stereological methods the authors still have to ensure that the sample areas are consistent between animals. The authors should indicate the anterior-posterior levels at which the three sections were taken and make sure that they were at the same level. They also should calculate the area that was sampled for each animal to make sure that there is no bias. How did the animals perform when repeating the two task conditions for the c-fos staining? Does repeating the task for a third time improve the behavior?

Ferguson et al. 2017 found that inhibiting the MD with the DREADD system in mice impaired initial acquisition and reversal learning but not ED set shifting using a different version of the Birrell & Brown odor based digging task. Parnaudeau et al. 2013 Neuron and 2015 Biological Psychiatry found a deficit in reversal learning but not in the initial acquisition of an operant based reversal learning task during DREADD-mediated MD inhibition. What explains the different outcomes between these two studies and the present study? Could it be related to inhibition restricted to the time window when the behavior is performed versus the chronic MD lesion? Alternatively, since animals had performed a reversal in the preoperative training, they are not naïve to it after the lesion, which may explain the absence of an effect of the lesion.

## Author Response

ID/ED eNeuro - reviewer’s comments

Dear eNeuro Editor,

We are pleased to resubmit for publication in eNeuro a revised version of our manuscript REF eN-NWR-0162-21, titled “Mediodorsal thalamus is critical for updating during extra-dimensional shifts but not reversals in the attentional set-shifting task”.

We appreciate the thoughtful comments received from the reviewers, and we are grateful for the opportunity to address their concerns through this revision. Reviewers expressed very helpful comments and indicated a number of opportunities for improving the manuscript - opportunities that we took very seriously and have incorporated into our manuscript.

We have implemented the changes needed to address all the comments we received. The enclosed memo describes, briefly, how we have modified the original draft to address the reviewers’ concerns. To facilitate the reviewing process, and to make future communication more efficient, each individual comment was identified and our responses are indicated in red. Our implementation of the changes that reviewers suggested changed some of the results that we reported in the original draft making some effects stronger.

We are convinced that our attempt to address the reviewers’ concerns resulted in a stronger and more compelling manuscript, and we are grateful for the opportunity to contribute to eNeuro. We are looking forward to hearing from you.

Sincerely,

The Authors

Synthesis Statement for Author (Required):

Thank you for your submission, the reviewers are largely in agreement that this is an interesting set of findings that add new knowledge to the literature. They have both included a series of points that each require being addressed, as set out below. I would also add that as the study appears to only have used male mice, please could this be stated in the abstract and could there be a sentence added to the discussion stating that the results may not generalise to females.

This has been done, and to clarify the animals are male rats.

Reviewer #1

This paper presents an interesting set of findings that MD lesions affect initial sensory discrimination (SD) and ED shifting of attention while sparing reversal learning and ID shifts of attention. These results are potentially important for understanding the role of rodent MD in cognitive flexibility. The experiments appear to be carefully conducted. The authors follow the well-established methods developed by Birrell and Brown (2000) to examine ID/ED attention shifting. MD lesions appear to be made skillfully and lesion extent analyzed quantitatively. C-Fos methods were used to assess neuronal activity in orbital and medial PFC 90 minutes following a second exposure to SD and ED training. These analyses lack necessary controls to determine whether MD lesions specifically affect the activity of these cortical areas during the behavioral tasks.

There are several issues that should be addressed in a revision of this paper.

1) As the authors note, Birrell and Brown found no effect of medial prefrontal lesions on initial SD. It is unclear why they predict that MD lesions should affect both SD and ED in the introduction (lines 73 and 74) while acknowledging that medial prefrontal lesions do not. This seems to be an important predicted distinction between MD and PFC function that should be addressed more directly.

Thank you for this comment. To clarify, our position is that the MD and mPFC are working together in partnership, contributing different but complementary roles to goal-directed behaviours. Therefore, we are not wanting to suggest that the MD mimics PFC behaviours. Rather, the MD has its own unique pattern of interconnected inputs from subcortical and cortical structures that identify it as an important nodal structure within a wider neural network that includes frontal cortex. Similarly, the mPFC also has its own set of inputs so together the unique and overlapping inputs associated with the two structures complement each other. Dr Mitchell’s work in non-human primates has helped to clarify this position as research from her lab has shown that the MD is not simply mimicking PFC damage. For example, the MD contributes to new learning but is not involved in retention of preoperatively learnt strategies or visual discriminations. In contrast, the PFC does contribute to learning and retention (e.g., Mitchell et al 2007a, b; Mitchell and Gaffan, 2008).

Thus, we predicted MD contributes to learning because the Mitchell lab has identified that the primate MDmc (MDm in rodents that was lesioned in the current set of rats) contributes to learning complex visual discriminations associated with reward (Mitchell and Gaffan, 2008; Browning et al 2015; Chakraborty et al., 2016; Chakraborty et al., 2019). In addition, MD lesion rats are impaired when learning discriminations associated with reward (Mitchell and Dalrymple-Alford, 2005; Hunt and Aggleton, 1998; Courtiol and Wilson, 2019; with Mitchell and Chakraborty, 2013 providing an overview table of the effects of MD lesions in animals). Ferguson and colleagues (2017) also showed perturbations to the MD impair learning discriminations. Further, while we have shown that the animals with MD damage are impaired in learning, this damage does not render the animals completely amnesic. Instead, what we and others have observed is that when given further trials, the animals are typically able to learn the discriminations, and this was certainly true for the current set of rats.

We have adjusted the text in the introduction in several places to explain our reasons for running this experiment and our hypothesis further. The text is coloured in red in the manuscript.

2) The authors ascribe the decrease in trials to criterion for REV3 for the MD lesion group to effects of overtraining. This begs the question of why a similar effect not observed for REV 1 following SD overtraining here or for REV3 following ED overtraining in Birrell and Brown? Are there other potential explanations for this result?

Thank you for highlighting that this explanation is not yet fully clear. In subsequent work, the Brown lab has identified that rats who are over-trained take less trials to complete reversals. (Dhawan SS, Tait DS, Brown VJ. More rapid reversal learning following overtraining in the rat is evidence that behavioural and cognitive flexibility are dissociable. Behav Brain Res. 2019 May 2; 363: 45-52. doi: 10.1016/j.bbr.2019.01.055.). This behavioral performance has been identified and studied for many decades in psychology (e.g. Reid, 1953; Lovejoy, 1966). Lovejoy, E. (1966). Analysis of the overlearning reversal effect. Psychological Review, 73(1), 87-103, doi: 10.1037/h0022687

We believe that the additional trials required by the MD lesion rats, relative to the MD Sham controls, to learn the new discrimination during the ED subtask caused an overexposure to all of the sensory dimensions. Therefore, the MD lesion group may have developed, through overtraining, a greater understanding of the rewarded and unrewarded stimulus dimensions. This may then have increased the salience of the predictive cues and reduced/eliminated the number of factors that can led to an error. Others have also shown similar effects in rats during studies to assess model based and model free learning (e.g. Nilsson et al., 2015, doi: 10.1016/j.neubiorev.2015.06.015 provides a good review).

Given our explanation of overtraining, we still would not predict to see an enhanced effect on REV1 as the SD (simple discrimination) subtask does not use both sensory dimensions. The two types of sensory discriminations (odour and digging medium) are only introduced for the CD (compound discrimination) subtask, which is run immediately after the SD subtask. REV1 follows the CD subtask. The rats did not experience overtraining on the CD task as they completed it in a similar amount of trials as the MD Sham controls. Throughout these two further subtasks (CD and REV1), the rats had to apply the strategy, (i.e. respond to the rewarded sensory dimension (either odour or digging media) that was learnt during the SD).

Finally, as indicated, we had expected the MD lesion rats to be impaired in reversal learning but this wasn’t the case. However, all the animals had been trained to perform the task preoperatively prior to their lesion (as also identified by Reviewer 2), so all the rats were familiar with the concept of reversal learning and knew that it could occur within the task structure (Costa et al, 2015: doi: 10.1523/JNEUROSCI.1989-14.2015 ; Jang et al 2015: doi: 10.1523/JNEUROSCI.1594-15.2015).

We attribute the differences between our results and the study of Birrell and Brown (2000) to the differences of the investigated areas. Birrell and Brown investigated the role of the medial prefrontal cortex in ID/ED. The lesions were centered on prelimbic cortex and included damage to infralimbic cortex. However, in the rat, the MD projects to medial prelimbic cortex, orbitofrontal cortex and cingulate (Cg1) cortex. A large body of research indicates that separate rat prefrontal cortex subregions differentially contribute to learning, memory, and other cognitive functions (for a review, see Dalley, Cardinal, & Robbins, 2004; Uylings, Groenewegen, & Kolb, 2003). Differential involvement of prefrontal cortex areas has been observed in tests that examine sustained attention, working memory, memory consolidation, and task switching (Birrell & Brown, 2000; Chudasama et al., 2003; Delatour & Gisquet-Verrier, 2000; Dias & Aggleton, 2000; Ragozzino et al., 2003; Rudebeck et al., 2006). For example, several previous studies showed that anterior cingulate lesions did not impair reversal learning in two-choice discrimination tests (Becker et al., 1981; Bussey et al., 1997; Chaillan et al., 1997; Joel et al., 1997; Meunier et al., 1991; Warburton et al., 1998). Gemma and colleagues (2006) showed a deficit in incorporating information about previous action outcomes to guide subsequent behavior. Lateral OFC inactivation preferentially impaired performance during reversal phases. In contrast, prelimbic inactivation caused an apparent improvement in performance by increasing the number of reversals completed. This was associated with enhanced sensitivity to recently rewarded actions and reduced sensitivity to negative feedback. Infralimbic inactivation had no effect, whereas the anterior cingulate appeared to play a permissive role in this form of reversal learning. Hence, we argue that the impairments and changes we report in the current study are the combination of the effects of the dysfunction of MD and its cortical targets.

We have updated our discussion to capture these main points.

3) The authors argue (paragraph beginning on line 529) that reduced correct 2nd choice responses represent a failure to adapt initial choice responses. Couldn’t this simply represent a failure to recognize a correct first choice (i.e. failure to acquire the discrimination rule) leading to an incorrect response to the alternative pot?

Thank you for this comment. However, we wish to highlight that during the ID/ED task, a correct choice is when the rat digs the bowl containing the right dimension. During the task we have noticed that the rats exhibited one of two behaviours before making a choice, they either visit the compartment containing the correct/rewarded dimension and dug to get the reward (correct choice), or visited the opposite compartment, explored the bowl without digging, then switched to the compartment containing the correct dimension and then dug. The latter is considered as a correct 2nd choice response. A failure to acquire the discrimination rule resulted in exploring and digging the incorrect bowl.

For this analysis, we are trying to capture what aspects of learning are impaired after permanent MD lesion. We agree with the proposition that the rats may have simply not correctly identified a correct first choice. However, we disagree with this as a complete explanation because if so, this would suggest the rats are perhaps slower to learn due to a sensory deprivation or are only perseverative with their responding. During learning, there are many strategies that could be used to acquire the new rule for optimal responding. We think that the MD, via thalamocortical interactions, is helping the cortex to compute the optimal signals as a consequence of the here and now within a given trial. With the MD gone, the PFC is still receiving multiple signals but the relevant ones are no longer identifiable. This may be the result of changes to coherence or in density of firing or via disruptions linked to excitation and inhibition in the PFC. So, while the rat is still able to learn and make decisions, it is not able to do so quickly and optimally in the here and now of a single trial. Thus, with the MD gone and this additional route of signal transfer no longer being conducted to the cortex, we think that the rats are not able to quickly learn to adapt after the task has changed (ED subtask) and instead it takes them longer to determine which stimulus dimension is now the correct one to guide optimal behavior. What we observe in their behavior is that the MD lesion rats return to sampling the unrewarded stimulus or previous location of the bowl because other brain regions had already directed the motor responses to go with this option at the start of the trial. We consider that the reduced correct 2nd choice responses represent a failure to adapt the initial choice response during a particular trial and thus a failure of within trial behavioural flexibility.

4) The authors argue (paragraph beginning on line 558) that reduced c-Fos counts provide evidence that “MD-PFC interactions contribute to learning and cognitive flexibility”. It is not clear what to make of the c-Fos results. Comparable decreases were observed after a 90 m period that followed second exposure to SD and ED. However, there are no controls for nonspecific effects of MD lesions. Would comparable decreases have been observed for a group of rats given MD lesions with no behavioral training? Are decreases observed in OFC and PrL greater than in other areas of cerebral cortex? Given the lack of such basic controls, what do the c-Fos results add to this paper?

We agree that sufficient controls have not be utilized for this part of the study so we have decided to remove the c-Fos imaging from the paper. We needed to have included a lesion and MD Sham group that sat in the cage but it was not possible for this study.

Reviewer #2

In this study the authors used excitatory lesions in the rat to study the impact of mediodorsal thalamus (MD) lesions on flexible behavior. Flexible behavior was tested in the Birrell & Brown odor-based rule shifting task that allows for assessing initial association, within perceptual dimension rule shifts (ID or reversal learning) and extradimensional rule shifts. Although the question addressed is not novel the data are important as they contradict previous findings and the discussion about these contradicting data will enhance our understanding of the role of the MD in flexible behavior. The experiments are well done. I have mostly comments about the selection and the interpretation of the data.

1) Figure 2: All animals completed the ID/ED task pre- and postoperatively. The authors should include both sets of data in Figure 2 so it can be seen how the animals performed pre-operatively. Also, I could not find in the method section that rats were tested pre- and postoperatively.

Thank you very much for pointing this out. We have now clarified in the methods that preoperative training occurred in the task. In the original draft, we had only mentioned preoperative training in the results section. For Figure 2, we have now included both preoperative and post-operative data.

2) The authors interpret the deficit in the initial SD acquisition as that the rats have to learn a stable response strategy, which is impaired in MD lesioned rats. Once the rats have learned the strategy MD lesions have no effect on the learning of switched or reversed associations (as long as they are in the same perceptive domain). I have a problem with this interpretation because the rats have already acquired the response strategy pre-operatively. Thus, performing SD acquisition is not new for the rats. Is it possible that the lesioned rats forgot about or are not able to implement the previously learned SD-response strategy?

It is true that the SD (simple discrimination) subtask is not new for the rats because they were run preoperatively on all subtasks of the ID/ED task in the same order as the postoperative test. However, they had not acquired the response strategy preoperatively to complete the SD postoperatively as the final subtask ran preoperatively was REV3, which has two sensory dimensions (odour and digging media, i.e. four stimuli). In contrast, the SD subtask uses only one sensory dimension (odour or digging media with two stimuli to discriminate), so the SD subtask is different for the animals to encounter after their surgery.

In the preoperative session, the SD is the first subtask the rats encounter. However, in the postoperative session while it is still the first subtask they encounter, it is following on from REV3 of the preoperative session and surgery (MD Sham or Lesion) and at least two weeks to recover and return to 85% of baseline weight. Given all of this, it is possible that the rats had forgotten about the strategy to conduct a simple discrimination but we disagree that this is the case as monkeys with MD lesions are still able to implement a previously learnt response strategy (Mitchell et al, 2007 J Neurosci). Instead, what we think is that the rats with MD lesions are unable to quickly update/change to a different response strategy (i.e. revert back to dealing with only one sensory dimension with two stimuli) in addition to rapidly learning this new sensory dimension discrimination. However, as indicated in response comment 1) to Reviewer 1, while the MD lesion rats were impaired in learning they were not densely amnesic and instead just needed more trials to acquire the correct response strategy.

3) I don’t understand the argument of overtraining for the improved performance of MD lesioned mice in REV3. First, it takes MD lesioned mice longer to reach criterion in the ED shift condition thus doing more trials is not necessarily an overtraining. Second, if the mice were indeed overtrained, I would have expected a deficit and not an improvement in performance.

Please see our above response [comments 2)] in reply to Reviewer 1, as we cover the explanation of overtraining there. In addition, to clarify, we were using rats in this research, not mice.

4) It is possible that the MD lesion animals never properly learned the rule. They may have been able to reach the criterion with a less firm understanding of the rule than the MD sham animals. In that case, they would also be quicker to learn the reversal as the rule that they would have to un-learn would not have as strong an association.

This is also a possible explanation, and one we cover in the manuscript (line 444 onwards). The standard version of the Birrell and Brown (2000) task that we used has been adapted, if rats are not able to learn the rule. This deficit in rule learning would be displayed with impaired performance during the CD, REV1, and ID shifts. In the adapted version of the task, additional ID shifts have been introduced so that the rats with profound learning deficits may be able to learn the rule prior to the ED subtask being introduced. This has been the case for rats with other thalamic lesions, namely the anterior thalamus (ATN: Wright et al 2015) and the nucleus reuinens (NRe: Linley et al 2016). However, rats with MD lesions can and did learn the rule. As indicated by their performance in the task, they were not impaired in the CD, REV1 or ID shifts. Thus, we propose that the learning deficit is one that is related to not being able to rapidly adapt and update behavioral responses (choices) during the trial as a consequence of the rule change rather than an overall deficit in learning per se.

5) Figure 3: The rational and interpretation for the analysis of the 2nd correct choice is not clear to me. As per provided definition, the rat does a correct 2nd choice when the first bowl it visited was an incorrect bowl and the rat didn’t dig into the incorrect bowl. In this scenario if the rat doesn’t dig in the first bowl, this is for me an indication that the rat already understands the new rule based on previous trial outcomes. It is not clear to me what the authors mean by learning on the fly and why the deficit cannot be explained by an enhancement in perseverative responding or an inability to respond to negative feedback as explained in line 350. To address perseverative responding the authors should analyze the percentage of perseverative vs non-perseverative errors (e.g., as in Ferguson et al. 2018 Biol Psych).

Any tendency to perseverate in responding to a previously correct stimulus would impair reversal learning. However, our MD rats do not show impairments during the reversals, i.e. they are not perseverating (like OFC lesion rats). Interestingly, OFC lesion rats (McAlonan and Brown, 2003) also had a preoperative test session that exposed them to the reversal but this didn’t mitigate the postoperative test impairment. The OFC lesion rats, in contrast, were not impaired in the ED shift. Thus, this ID/ED task, allows for different types of behavioral flexibility and cognitive control to be assessed within the same session.

Reversals in this task are a special case of an intra-dimensional (ID) shift. Like the ID shift, reversal learning does not require attention to be reoriented, because the attended dimension remains the same as the previous subtasks (SD, CD). However, unlike the ID shift in which all the exemplars are novel, in the reversal, the exemplars remain the same as the just completed subtask (CD for REV1 and ID for REV2): the previously correct exemplar is now incorrect, and the rat must respond to the previously incorrect exemplar.

Therefore, given that the rats are not showing perseverative responding during reversal learning, it suggests that the ED shift deficit for our MD lesion rats is not caused by a problem with perseverative responding either (although rats may make perseverative responses) because the rats are as able as controls to abandon a response to a previously reinforced stimulus. Rather, the rats were unable to learn quickly, within the trial, if they had chosen the incorrect bowl to dig in and instead, continue to dig in the incorrect bowl (i.e. they made less 2nd choice correct responses than the MD Sham controls).

It was not possible to divide performance into epochs corresponding to a period of perseverative responding and a period of learning (Ferguson et al. 2018 Biol Psych; Hunt and Aggleton, 1998), because learning was rapid in the control group, meaning that there were not enough trials. In order to reach criterion, the rats must complete 6 consecutive correct trials. In the MD lesion group, during the ED subtasks, we observed that the rats would sometimes pick two or three correct bowls in a row and then switch to the other (incorrect) bowl for a couple of trials and then back and forth. In monkeys with MD lesions, we have also observed that they don’t perseverate in their responding (Mitchell et al., 2007; Chakraborty et al. 2016). Instead, MD lesion monkeys adopt a strategy that causes an increase in switching their choice responses between all the available options (Chakraborty et al. 2016). We were able to conduct this additional analysis with the monkeys as they were completing 300 trials per day. This type of behavioral responding suggests that the monkeys and rats are aware of the task structure but they are requiring many more trials to be able to develop a hypothesis about the optimal stimuli that will provide the reward most often.

6) There are alternative explanations for less correct 2nd choices. First, it possible that lesioned animals do less correct 2nd choices because they less often approached the incorrect bowl as the first bowl. Second, lesioned animals do less correct 2nd choices because they do more mistakes. By digging more often into the 1st bowl, when it is incorrect, they have less possibilities to do a 2nd correct choice.

The left-right position of presentation in the cage was a pseudorandom series determined in advance. The trials are forced choice so they have to respond to one of the bowls. They are motivated to complete the task and receive their daily food after testing. Their weight is reduced to 85% of free-feeding weight and all the rats readily ate the Honey Nut Cheerios cereal. The criterion to complete each subtask is 6 consecutive correct choices. All animals had the same opportunity to approach the correct and the incorrect bowls. When the rats made a mistake by digging in the incorrect bowl, this was scored as error not a correct 2nd choice response. Because lesioned animals make more errors, we evaluated their performance as percentage of correct 2nd choice responses out of the total number of correct choices (please see Fig.3).

7) Would it be easier for the animal to make the assessment of what is in each pot for trials where odor is the rule as opposed to texture? If odor is the dimension that is relevant, the animal might be able to smell the odor from a distance without approaching the pot and make a “2nd choice” without having to go visit both pots.

This is a good question to explore. We’ve conducted additional analyses to investigate this. We have included these details in the results section from line 361 onwards.

We analyzed the rats’ errors made reaching criterion during the ED subtask, as a consequence of whether they experienced a sensory dimension shift from odor to digging medium or digging medium to odor, which indicated that the increase in errors occurs irrespective of the direction of the attentional shift. The univariate ANOVA with Lesion_type and Sensory Shift as the between-subject factors and errors to criterion as the dependent measure revealed a main effect of Lesion_type, F(1, 21) = 106.03, p < 0.001, but no main effect of Sensory Shift, F(1, 21) = 3.84, p= 0.063 and no interaction, F(1, 21) = 0.19, p=0.666.

8) Figure 4: Reaction time seems not the appropriate term here as reaction times are usually in the range of milli-seconds. I would rather suggest to use the term “latency to dig”. Why is this measure only shown for error trials? It should be shown for both. The longer latencies to dig in the lesioned animals could be an indication of a change in motivation. This should be discussed.

Thank you for this suggestion, we have adjusted the figures to show “latency to dig” instead of “reaction time”. We have not included latencies on correct choices as this is a forced choice task with a criterion of 6 consecutive correct responses indicating acquisition in the task. All rats completed the correct trials well. Thus, we have only included latency to dig for the error trials to be able to highlight that all the rats were motivated to perform the task and this wasn’t the reason for their deficit. While the MD lesion rats were slower to respond on error trials during the first three subtasks (SD, CD and REV1), they were not unmotivated to perform the task, rather they were attending, learning the rewarded sensory dimension more slowly, but actively engaged in each of the subtasks and overall testing session.

9) Line 429: “The analysis of behavioral responses........is critical for monitoring and updating of choice response rules”. Again, I don’t see how the 2nd choice responses support this interpretation.

When the rats made more 2nd choice responses that means that they could adapt and change their response within a trial according to the environmental contingencies via monitoring and updating their choice response.

We have adapted this sentence now on line 415 onwards as indicated below.

The analyses of behavioral responses indicated that the MD lesion rats made significantly fewer correct 2nd choice responses within a trial suggesting that information transfer via the MD is critical for the rapid, within trial monitoring and updating of an optimal choice response.

10) Figure 5-7: Even if the authors did not used unbiased stereological methods the authors still have to ensure that the sample areas are consistent between animals. The authors should indicate the anterior-posterior levels at which the three sections were taken and make sure that they were at the same level. They also should calculate the area that was sampled for each animal to make sure that there is no bias. How did the animals perform when repeating the two task conditions for the c-fos staining? Does repeating the task for a third time improve the behavior?

We have removed the c-fos imaging and analyses from the manuscript as there are insufficient control data.

11) Ferguson et al. 2017 found that inhibiting the MD with the DREADD system in mice impaired initial acquisition and reversal learning but not ED set shifting using a different version of the Birrell & Brown odor based digging task. Parnaudeau et al. 2013 Neuron and 2015 Biological Psychiatry found a deficit in reversal learning but not in the initial acquisition of an operant based reversal learning task during DREADD-mediated MD inhibition. What explains the different outcomes between these two studies and the present study? Could it be related to inhibition restricted to the time window when the behavior is performed versus the chronic MD lesion? Alternatively, since animals had performed a reversal in the preoperative training, they are not naïve to it after the lesion, which may explain the absence of an effect of the lesion.

In response to the Parnaudeau et al. 2013 Neuron and 2015 Biological Psychiatry studies, during both the lever press operant box task and the T-maze task, the initial acquisition of the correct discrimination was delayed for the mice with the DREADDs activated (e.g. please see Figure 3B discrimination and Figure 4 from the Neuron paper). The comparison they made in Figure 3B was for the trials conducted on session 7 of learning only. In addition, the volume of DREADDs that were infused into the mice were extensive (0.5 μl per hemisphere - overall total of 1 μl in each mouse). By comparison in our rats’ brains in the current study and previous studies conducted during my PhD (Mitchell and Dalrymple-Alford 2005, 2006), we infused (0.20 μl in the anterior MD and then 0.18 μl in the posterior MD - overall total of 0.38 μl per rat). From my extensive experience of conducting infusions into the dorsal thalamus (ATN, MD) in rodents and monkeys, the use of this larger volume in mice would mean that the DREADDs had almost certainly spread to adjacent dorsal thalamic structures (likely the ANT, intralaminar nuclei (ILn), and potentially downwards to the nucleus reuniens (NRe)). All of these adjacent structures are also interconnected to the medial PFC in rodents. Thus, after i.p. CNO injections all areas (e.g. the MD and ATN, ILn, NRe) that received the DREADDs would be inactive, which would create widespread temporary dysfunction across several neural networks. The larger volume of DREADDs used in these studies was also suggested as a difficulty for interpretation by Wolff and Vann (2019, J Neurosci). Thus unfortunately, from the Parnaudeau and colleagues’ studies, it is difficult to be able to conclude that the performance deficits they observed were only caused by inhibition within the MD. In addition, typically permanent perturbations to the MD do not cause deficits in working memory for rats, monkeys or humans so the Parnaudeau results are not similar to what is observed in other studies either.

For the Ferguson et al (2017) study, the initial deficit in acquisition after MD perturbations is similar to what we observed in our ID/ED task - we have now included this reference in our introduction. However, we suggest the difference from our results for the reversal learning is that the reversal tasks used across ours and their studies were not the same. For example, the Ferguson et al task consisted of an initial association phase where the odour was always rewarded followed with a rule shift phase where the media was always rewarded, then the reversal was performed (the previously unrewarded media is therefore rewarded), which we think made it difficult for the mice to get the associations sorted during the reversals - potentially a deficit in updating reward associations (which rats and monkeys with MD lesions are impaired at doing). Thus, the mice may not have learnt the required strategy to get the task sorted as they were performing concurrent associative learning. In our task, the rats initially learnt which sensory dimension was correct (similarly impaired in acquisition as the Ferguson mice are too) but they went on to learn concurrent discriminations applying a response strategy that involved the same sensory dimension. Thus, the task structure and current strategy is key to performing in the task, as is a core difference between the two types of reversal learning.

We have adapted the discussion to incorporate these points.
