# Supplementary figures and images for: Mediodorsal Thalamus Is Critical for Updating during Extradimensional Shifts But Not Reversals in the Attentional Set-Shifting Task
Source: eNeuro. 2022 Mar 3;9(2):ENEURO.0162-21.2022. doi: 10.1523/ENEURO.0162-21.2022 (PMC8906789; doi:10.1523/ENEURO.0162-21.2022)

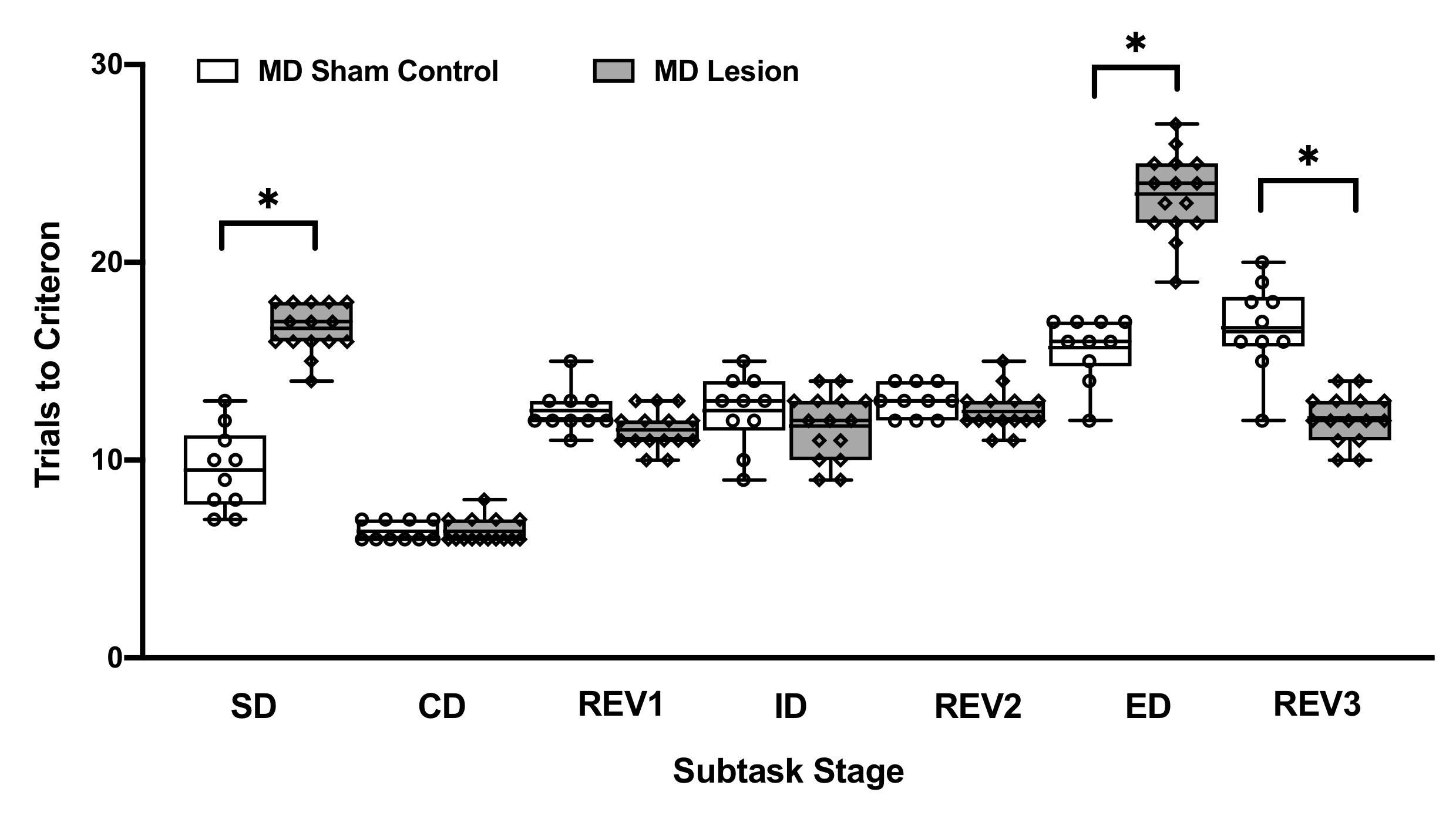

Supplement: Extended Data Figure 2-1 — Boxplots of the mean trials to criterion for individual rats during the postoperative session of the attentional set-shifting task. MD lesion rats took significantly more trials to learn the SD and the ED subtasks compared to MD sham controls (*p < 0.001). MD sham control rats also needed more trials to learn the ED, as expected. The MD lesion rats required significantly fewer trials to learn REV3, the reversal occurring immediately after the ED, compared to the MD sham controls (*p < 0.001). Box shows 1st and 3rd quartile and whiskers are the minimum and maximum trials to criterion for individual rats. Download Figure 2-1, TIF file. [file enu-eN-NWR-0162-21-s02.tif]
